# Emerging role of IκBζ in inflammation: Emphasis on psoriasis

**DOI:** 10.1002/ctm2.1032

**Published:** 2022-10-17

**Authors:** Preeti Gautam, Sylvain Maenner, Frédéric Cailotto, Pascal Reboul, Stéphane Labialle, Jean‐Yves Jouzeau, Frédéric Bourgaud, David Moulin

**Affiliations:** ^1^ Laboratoire IMoPA UMR 7365 CNRS‐Université de Lorraine, Biopôle de l'Université de Lorraine Vandœuvre‐lès‐Nancy France; ^2^ Temisis Therapeutics Vandœuvre‐lès‐Nancy France

**Keywords:** psoriasis, ikappabZeta, IκBζ, inflammation, *NFKBIZ*

## Abstract

Psoriasis is a chronic inflammatory disorder affecting skin and joints that results from immunological dysfunction such as enhanced IL‐23 induced Th‐17 differentiation. IkappaB‐Zeta (IκBζ) is an atypical transcriptional factor of the IκB protein family since, contrary to the other family members, it positively regulates NF‐κB pathway by being exclusively localized into the nucleus. IκBζ deficiency reduces visible manifestations of experimental psoriasis by diminishing expression of psoriasis‐associated genes. It is thus tempting to consider IκBζ as a potential therapeutic target for psoriasis as well as for other IL23/IL17‐mediated inflammatory diseases. In this review, we will discuss the regulation of expression of *NFKBIZ* and its protein IκBζ, its downstream targets, its involvement in pathogenesis of multiple disorders with emphasis on psoriasis and evidences supporting that inhibition of IκBζ may be a promising alternative to current therapeutic managements of psoriasis.

## Graphical abstract

Expression of *NFKBIZ* is stimulated by cytokines such as IL‐17, TNFα, IL‐1β and IL‐36. Increased expression of IκBζ eventually activates NF‐κB leading to increased expression of various proinflammatory cytokines, chemokines, as well as psoriasis‐associated genes. This cascade of molecules causes recruitment of neutrophils and other immune‐mediated inflammatory cells, which consequently leads to the development of psoriasis‐like skin inflammation.

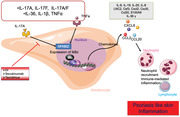

## 1 INTRODUCTION

Psoriasis is a common chronic inflammatory disease characterized by relapsing cutaneous erythro‐squamous patches (psoriasis vulgaris) and/or inflamed joints (psoriatic arthritis). Psoriasis affects millions of individuals worldwide with a prevalence ranging from 0.5% to 11% in adults and below 1.37% in children.^1^ Even though its etiology still remains elusive,^2^ the implication of the immune‐immediate response engaging Th1, Th17, γδ T cells, dendritic cells and keratinocytes has been described to play a significant role in disease immunopathogenesis.^3^, ^4^, ^5^

In general, IL23 cytokine induces the secretion of IL17 by skin Tγδ cells, mucosal‐associated invariant T cells, innate lymphoid cells, and possibly by neutrophils leading to the induction of the inflammatory response.^6^ Therapeutics targeting IL23 and IL17 have been clinically approved and found to significantly manage the disorder.^7^ In addition to psoriasis, IL23/IL17 axis is a global immune regulatory mechanism involved in the pathogenesis of multiple immune‐mediated inflammatory disorders such as spondyloarthritis,^8^ rheumatoid arthritis,^9^ Crohn's disease,^10^ and ulcerative colitis.^11^

The protein IkappaBZeta (IκBζ) was independently discovered by Kitamura et al., Shiina et al. and Haruta et al.^12^, ^13^, ^14^ It was initially named as “molecule possessing ankyrin‐repeats induced by lipopolysaccharide” (MAIL)^12^ and “interleukin (IL)‐1‐inducible nuclear ankyrin‐repeat protein” (INAP).^14^ For sake of clarity, in the present manuscript, *NFKBIZ* (nuclear factor of kappa light polypeptide gene enhancer in B cells inhibitor, zeta) and IκBζ are used hereafter to refer to gene/mRNA and protein, respectively. IκBζ belongs to the nuclear IκB family and carries ankyrin repeats‐containing domains. In mice, stimulation of the NF‐κB inflammatory response by intraperitoneal injection of lipopolysaccharide (LPS) rapidly induced expression of *NFKBIZ* mRNA in the spleen, lymph nodes and lungs which further potentiated interleukin‐6 (IL‐6) mRNA expression.^12^ Moreover, IκBζ was found to migrate promptly into the nucleus to regulate NF‐κB activity,^14^, ^15^ a key inflammatory pathway involved in psoriasis onset. Interestingly, recent studies have highlighted the link between IκBζ and psoriasis in both in vivo and in vitro psoriatic conditions.^16^, ^17^, ^18^, ^19^ With the aim to formalize the contribution of IκBζ to psoriasis via NF‐κB modulation, we reviewed the current knowledge on the *NFKBIZ* gene including its expression regulation, its genetic variations, its encoded protein IκBζ and how this latter associate with NF‐κB to modulate multiple downstream targets. We also conclude on the recent advances highlighting its implications in immune homeostasis related to several pathologies such as psoriasis but also cancer and infections.

### 1.1 Generalities about the *NFKBIZ* gene and its protein IκBζ

By genomic mapping, Shiina et al.^13^ found that the *Nfkbiz* gene is located on the chromosome (Chr) 16 C1.2‐C1.3 and Chr 11q21.1 in mice and in rat, respectively. This gene is well conserved in human, chimpanzee, rhesus monkey, dog, cow, as well as in mouse, chicken, rat and zebrafish.^20^

The mice *Nfkbiz* gene encodes for three isoforms of IκBζ protein, the longest isoform 1 composed of 728 amino acids (AA) named as IκBζ(L)^12^; the shorter isoform 2 of 629AA lacking the first 99AA named IκBζ(S)^21^; and the isoform 3 of 534AA lacking the AA 236–429 named as IκBζ(D).^22^

**FIGURE 1:**
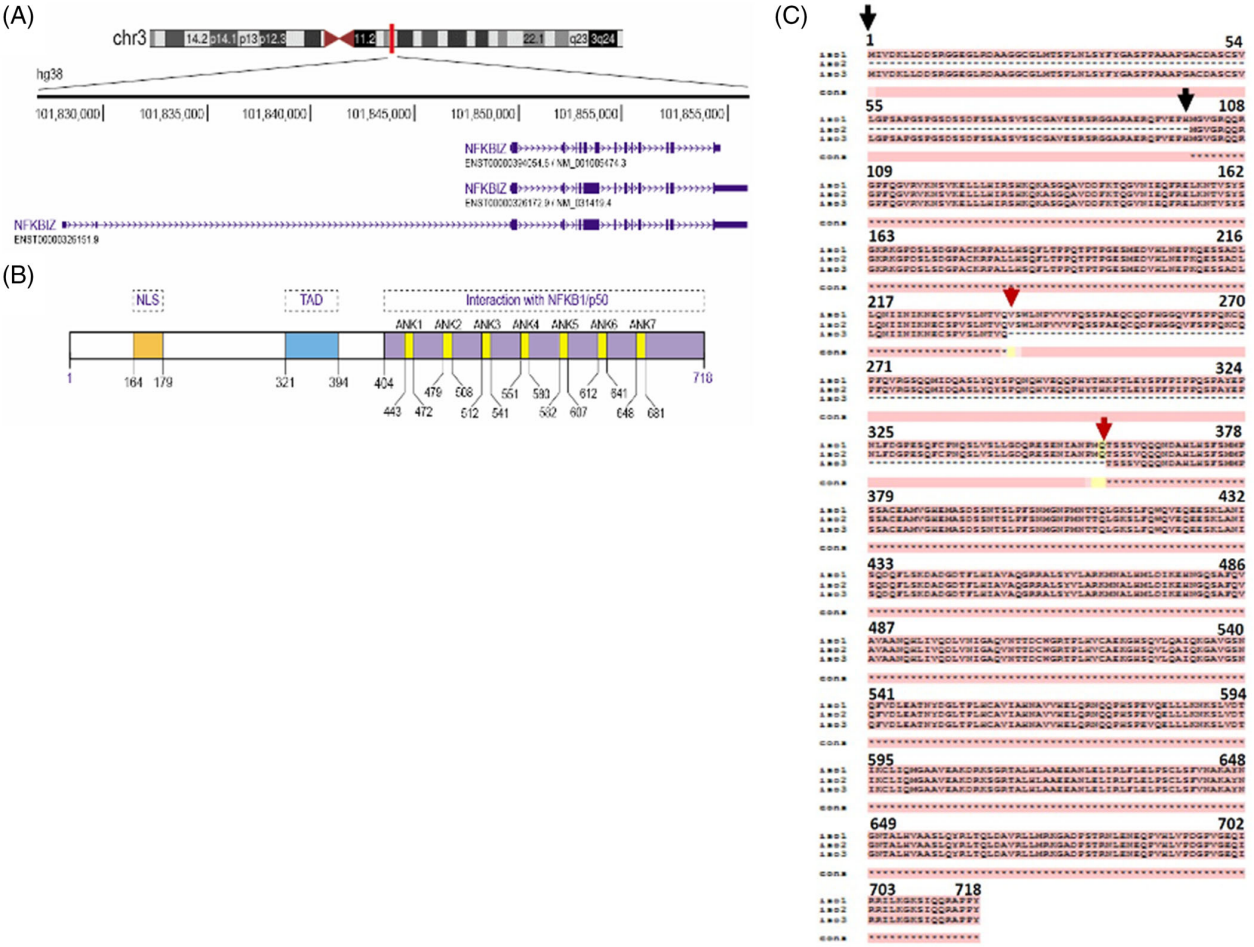
Schematic representation of the chromosomal localization and transcript variants (demonstrated using https://genome.ucsc.edu/) (A), protein structure of human IκB‐ζ (B), sequence alignment of three human isoforms (performed with https://www.ebi.ac.uk/Tools/msa/tcoffee/) (C). NLS, nuclear localizing signal; TAD, transactivating domain; ANK, ankyrin‐repeat; NF‐κB/p50, nuclear factor of κB; NFKBIZ, nuclear factor of kappa light polypeptide gene enhancer in B cells inhibitor, Zeta

The human *NFKBIZ* gene is a single copy gene mapped on the chromosome 3 at the locus 3q12.3 (Gene ID: 64332) (Figure 1A). *NFKBIZ* gene encodes for IκBζ protein with three isoforms produced by alternative splicing: the longest isoform 1 composed of 718 amino acids (AA) (encoded by ensembl transcript variant: ENST00000326172.9, NCBI Transcript ID: NM_031419.4) named as IκBζ(L); the shorter isoform 2 of 618 AA lacking the first hundred AA (represented with black arrow, encoded by ensembl transcript variant ENST00000394054.6, NCBI Transcript ID: NM_001005474.3) named IκBζ(S); and the isoform 3 long to 596 AA lacking the AA 237 to 358 (represented with red arrow, encoded by ENST00000326151.9) named as IκBζ(D) (Figure 1A,C). The amino acid sequence from 359 to 718 is conserved between the three human isoforms of IκBζ. Seven ankyrin repeats located in the carboxyl terminal domain of the protein were identified to interact with the p50 subunit of NF‐κB. Furthermore, several functional domains have been identified in the IκBζ(L) isoform: a nuclear localization signal (NLS) spanning 164–179 AA; an internal fragment between 321 and 394 AA with a transcriptional activity domain (TAD) (Figure 1B).^20^, ^22^ As isoform IκBζ(D) carries deletion in the central region supporting transactivation activity, this isoform may lack transcriptional activity.

### 1.2 Genetic variations in *NFKBIZ*

Several genetic variants have been reported in *NFKBIZ*. The presence of a 23 bp indel variation (rs3217713) in the intron 10 region of *NFKBIZ* was directly associated with psoriasis^23^, ^24^ and the patient's response to anti‐TNF drug adalimumab.^25^ In addition to psoriasis, the deletion allele of rs3217713 was also reported as an independent risk factor for the development of early‐onset coronary artery disease.^26^ This common indel polymorphism is positioned 3’ to exon 10 and co‐occurrence of alternative transcript lacking exon 10 predicts the possible impact of this genetic variant on pre‐mRNA splicing. As exon 10 encodes the ankyrin repeats of the protein which is responsible for the binding of IκBζ to NF‐κB/p50, this genetic variant might be considered as a potential marker for NF‐κB‐associated pathologies.^23^ Additionally, the rare genetic variant rs7152376 C in *NFKBIZ* was found to be more frequent in psoriatic arthritis patients in comparison to healthy controls.^27^ Chapman et al. and Sangil et al. also revealed association of multiple *NFKBIZ* genetic variants with invasive pneumococcal disease.^28^, ^29^ Despite the prominent contribution of *NFKBIZ* in inflammatory disorders, detailed studies elucidating the functional impact of its genotypic variants are lacking.

### 1.3 NF‐κB and IκBζ interaction

NF‐κB plays a central role in inflammation, cell growth, survival and differentiation. In resting conditions, NF‐κB is seized inside the cytoplasm by associating with IκB family proteins such as IκBα, IκBβ and IκBε. However, in the presence of stimulus such as bacterial LPS, cytoplasmic IκB proteins undergo phosphorylation‐induced degradation by the proteasome. The liberated NF‐κB translocates into the nucleus and activates the expression of several pro‐inflammatory cytokines, chemokines and anti‐microbial peptides, thereby playing a crucial role in host defense. IκBζ is barely present in resting conditions, but in the presence of stimulating agents like LPS, IκBζ is induced and localized into the nucleus where it preferentially interacts with p50 but not p65 subunit of NF‐κB.^30^, ^31^

IκBζ has been initially described as a negative regulator of NF‐κB, as IκBζ expression plasmid transfected into RAW264.7 cells was found to inhibit activity of NF‐κB reporter plasmid (pELAM1‐Luc) stimulated with LPS.^21^ This description of a negative activity of IκBζ on NF‐κB transactivation appears controversial, since the same author and others claimed thereafter the occurrence of latent transcriptional activation led by IκBζ upon interaction with p50 subunit of NF‐κB.^22^, ^32^ Afterward, Trinh et al. highlighted the functional interaction between NF‐κB and IκBζ.^33^ They demonstrated in cellulo, by pull‐down approach, that IκBζ, p50 homodimer of NF‐κB and DNA form a stable ternary complex, in which glycin‐rich region at the C‐terminal end of NF‐κB p50 homodimer binds with IκBζ, to prevent the proteolytic degradation of the NF‐κB subunit. This direct interaction between IκBζ and NF‐κB was shown to be a prerequisite for the expression of the pro‐inflammatory cytokine IL‐6 in activated peritoneal macrophages.^33^ Lately, Kohda et al. described that Asp‐451 in the N‐terminus of the ankyrin repeat 1 of IκBζ was critical for its interaction with the p50 subunit of NF‐κB, while the Lys‐717 and Lys‐719 in the C‐terminal region of ankyrin repeat 7 is responsible for IκBζ binding to the promoter of the lipocalin 2 gene leading to its transcriptional activation.^34^

### 1.4 Tissue expression and transcriptional regulation of *NFKBIZ*

Northern‐blot analysis revealed that *Nfkbiz* was barely expressed in unstimulated macrophages. However, upon proinflammatory challenge with LPS and IL‐1 β, expression of IκBζ was strongly induced. In mice, *Nfkbiz* mRNA level was significantly augmented in lung, liver, kidney, heart, testis, thymus, lymph node and spleen after intraperitoneal injection of LPS.^12^, ^21^ The basal expression of IκBζ was also reported in unstimulated cells within connective tissues like keratinocytes, corneal epithelial cells, conjunctival epithelial cells, few subconjunctival cells, tracheal epithelium and small intestine (and https://www.proteinatlas.org/ENSG00000144802‐NFKBIZ/tissue). Yamamoto et al. further demonstrated that in addition to IL‐1β and LPS, expression of *Nfkbiz* mRNA was also induced by peptidoglycans via TLR2, bacterial lipoproteins via TLR1/TLR2, flagellin via TLR5, MALP‐2 via TLR6/TLR2, R‐848 via TLR7 and CpG DNA via TLR9. Surprisingly, no expression of IκBζ was reported when cells were stimulated with TNFα alone.^37^ Once the above‐mentioned ligands bind to Toll/IL‐1 receptors, several signalling pathways are activated via the adaptor protein MyD88 and TRAF6. Eventually, TRAF6 activation leads to the stimulation of the MAP3K7/TAK‐1 complex which subsequently activates the NIK/IKK/IκB/NF‐κB pathway.^20^, ^38^ Then, NF‐κB acts as a transcription factor for many inflammatory genes which interestingly includes IκBζ since inhibition of NF‐κB's activities or invalidation of the MyD88 gene led to a complete repression of IκBζ expression in fibroblast cells, for instance.^37^, ^39^ Additionally, the sequence analysis of mouse *Nfkbiz* revealed a potential transcription factor binding site for NF‐κB as well as the existence of a TATA box element located into the proximal promoter region. Moreover, under the stimulus of LPS, the upstream region of the mouse *Nfkbiz* promoter was capable of promoting gene expression.^13^

### 1.5 Post‐transcriptional regulation of *NFKBIZ*

Activation of IL‐17, LPS and IL‐1β signalling pathways induces not only the transcriptional activation of *NFKBIZ* but also the stabilization of its mRNA. MaruYama et al. elucidated the contribution of LPS/IL‐1β‐MyD88 pathway to the stabilization of IκBζ mRNA.^40^ They showed that in response to LPS/IL‐1β stimulation, MyD88‐deficient macrophages failed to express *NFKBIZ* due to lack of stability of its mRNA. Moreover, IL‐17 also contributes to the stabilization of *NFKBIZ* mRNA.^41^ Mechanistically, IL‐17 induces expression of the RNA‐binding protein AT‐rich interactive domain containing protein 5a (Arid5a), which is recruited to the NF‐κB activator 1 (Act1) and TNF receptor‐associated factor 2 (TRAF2) complex. Herein, Arid5a performs two post‐transcriptional functions: first, it binds to 3’ UTR of *NFKBIZ* and counteracts the endonuclease Regnase 1‐mediated degradation resulting in mRNA stability; second, Arid5a facilitates translation of *NFKBIZ* by coordinating with eukaryotic translation initiation complex.^42^ Regnase‐1, also known as MCPIP1, degrades mRNA transcripts undergoing active translation following IL‐17 response, including *IL‐6* and *NFKBIZ*.^43^ Interestingly, a recent study reported that Regnase‐3, also known as MCPIP3, contributes to skin inflammation by directly degrading *NFKBIZ* mRNA.^44^

### 1.6 Downstream targets of IκBζ

In the last two decades, IκBζ has emerged as a critical regulator of NF‐κB‐mediated genes associated with inflammatory disorders. As stated earlier, IκBζ acts as a transcriptional regulator for various genes involved in cell survival, apoptosis and senescence. Interestingly, IκBζ itself lacks DNA binding site and rather assists other transcription factors in doing so.^20^ Some reports also hint that IκBζ negatively regulates STAT3 transcriptional activity (signal transducer and activator of transcription 3), thereby, influencing cellular growth and apoptosis.^45^ Furthermore, p38 mitogen‐activated protein kinase (MAPK), Act1 and Jun NH2‐terminal kinase (JNK) were also demonstrated as key signalling pathways in NFKBIZ/IκBζ regulation (Table 1).^46^

**TABLE 1:** Downstream targets of IκBζ with the respective stimulus

Target genes/proteins	Stimuli	Cells	Refs
IL‐6	LPS; pneumococcal strain D39	Swiss 3T3 cells; human monocytes; peritoneal macrophages	^6^, ^45^, ^46^
LCN2/ NGAL	IL‐1β; TNFα and IL‐17 costimulation	Epithelial cells; lung epithelial A549 cell line	^40^, ^41^, ^42^
IFNG	IL‐18 and IL‐1β acting in synergy with TNFα IL‐12/IL‐18	KG1 cell line Human NK cells	^43^, ^44^
DEFB4/ hBD‐2	IL‐17A	Normal human bronchial epithelial cells	^39^
IL‐36γ	IL‐17A	Psoriatic keratinocytes	^47^
CCL2 (MCP‐1)	LPS or bacterial peptidoglycan	In vivo zymosan peritonitis model	^48^
IL‐8	γ‐irradiation	Glioma cells	^49^
CXCL1	γ‐irradiation	Glioma cells	^49^
IL‐19	IL‐17A and TNFα	Human keratinocytes	^53^
IL‐20	IL‐17A and TNFα	Human keratinocytes	^53^
IL‐33 dependent cytokines and chemokines such as IL‐6, IL‐13, CCL2, CCL3, and TNFα	IL‐33	Bone marrow‐derived mast cells	^54^

CCL, chemokine (C‐C motif) ligand; Cxcl, chemokine (C‐X‐C motif) ligand; DEFB4, defensin beta 4; hBD‐2, human beta‐defensin 2; IFNG, interferon gamma; IL, interleukin; LCN2, lipocalin 2; LPS, lipopolysaccharide; MCP, monocyte chemoattractant protein; NGAL, neutrophil gelatinase‐associated lipocalin; TNFα, tumour necrosis factor‐alpha.

Numerous studies have highlighted that IκBζ binds to NF‐κB and upregulates the transcription of several secondary response genes such as *IL6*, *IL12*, *LCN2*, *IFNG* and defensin beta 4 *(DEFB4*). *DEFB4A* gene encodes for protein human beta‐defensin 2 (hBD‐2) which is primarily produced by epithelial cells after exposure to gram‐negative bacteria, viruses and pro‐inflammatory cytokines such as IL‐1β and TNFα.^47^ A study carried out by Kao et al.^48^ demonstrated that IL‐17A‐induced up‐regulation of IκBζ increases expression of *DEFB4*, since IκBζ knockdown reduced *DEFB4* expression in normal human bronchial epithelial cells. Neutrophil gelatinase‐associated lipocalin (NGAL) another epithelial cells associated protein, is encoded by the *LCN2* gene and is induced by IL‐1β during inflammation in lungs and colon, in a NF‐κB‐dependent manner.^49^, ^50^ Karlsen et al. indicated that co‐stimulation of epithelial cells with TNFα and IL‐17 led to IκBζ accumulation, which in turn bound to NF‐κB on the *LCN2* promoter, stimulating the expression of *NGAL* gene.^51^ Moreover, in lung epithelial A549 cell line, IL‐1β stimulation, but not TNFα, led to the transcriptional activation of NGAL which was also mediated by the binding of IκBζ to NF‐κB.^50^ Additionally, IκBζ also up‐regulates IFN‐γ in a NF‐κB‐dependent manner in human NK cells and KG1 cell line. Thus, IL‐18 and IL‐1β acting in synergy with TNFα were found to stimulate IκBζ‐mediated IFN‐γ expression in KG1 cells, and stimulation with IL‐12/IL‐18 was found to be sufficient to induce IκBζ which further results in secretion of IFN‐γ in human NK cells.^52^, ^53^ Pioneer studies carried out by Kitamura et al. revealed that upon LPS stimulation, IκBζ amplifies expression and secretion of IL‐6 in Swiss 3T3 cells.^12^ Mechanistically, TLR and NOD‐like receptor activation by several ligands (like LPS) is followed by the binding of IκBζ to p50 subunit of NF‐κB which results in remarkable production of IL‐6 in human monocytes.^54^ Sundaram et al. also demonstrated that under exposure to pneumococcal strain D39, IκBζ was induced in a concentration‐dependent manner in human monocytes but not in bronchial epithelial cells, consequently accounting for the increased expression of IL‐6 and GMCSF.^55^ IκBζ was also reported to regulate the production of IL‐36γ in psoriatic keratinocytes.^56^ Moreover, the role of IκBζ in the production of chemokines such as CCL2 (also known as MCP‐1), which is responsible for the migration of blood monocytes to the site of inflammation, was also shown. It was demonstrated that IκBζ‐deficient macrophages had an impaired secretion of CCL2 when challenged with LPS or bacterial peptidoglycan, whereas IκBζ‐deficient mice displayed a reduced CCL2 secretion and monocytes infiltration in the zymosan peritonitis model.^57^ Upon γ‐irradiation of glioma cells, expression of IκBζ was elevated which eventually led to enhanced transcription of tumour‐promoting cytokines such as IL‐6, IL‐8 and chemokine (C‐X‐C motif) ligand 1 (CXCL1).^58^ IL‐19 and IL‐20, members of the IL‐10 family, were found to be associated with psoriasis‐like skin abnormalities and upregulate psoriasis‐related cytokines.^59^, ^60^, ^61^ The knowledge on IκBζ regulation of inflammatory cytokines and chemokines was further extended in a study demonstrating that in human keratinocytes, synergistic induction of IL‐17A and TNFα regulates IL‐19 and IL‐20 mRNA and protein expression, by IκBζ‐mediated p38 MAPK, NF‐κB and JNK1/2‐dependent signalling pathway.^62^ In a recent study, NF‐κB‐mediated induction of IκBζ was found to boost the expression of IL‐33‐dependent cytokines and chemokines such as IL‐6, IL‐13, CCL2, CCL3 and TNFα in bone marrow‐derived mast cells.^63^ Overall, numerous evidences show that IκBζ in association with NF‐κB upregulates production and secretion of various pro‐inflammatory cytokines and chemokines in immune cells, epithelial cells and human keratinocytes. Thus, positioning IκBζ as a consistent player in the pathogenesis of skin inflammatory disorders such as psoriasis appears to be a particularly relevant hypothesis.

### 1.7 IκBζ in psoriasis

Psoriasis is characterized as a chronic immune‐mediated skin disease primarily provoked by increased expression of the pro‐inflammatory cytokines IL‐23, TNF‐α and IL‐17.^64^ Precisely, TNFα and IL‐17A have been considered as major players in the pathogenesis of psoriasis, since Chiricozzi et al. identified that costimulation of keratinocytes with TNFα and IL‐17A leads to synergistic upregulation of hundreds of genes, including a group of genes with the highest level of expression in psoriatic skin, such as *IL‐8, IL‐17C, IL‐19, CCL20* and *DEFB4*.^65^ Although the underlying molecular mechanism is still not fully comprehended, these data highlight the potential relevance of the anti‐psoriasis therapeutics based on TNFα or IL‐17 antagonists. In this line, a significant step ahead was provided by an enhanced meta‐analysis and replication studies which allowed to discover a new psoriasis susceptibility locus. Herein, the *NFKBIZ* gene was reported as a downstream target of IL‐17 signalling in human skin keratinocytes.^17^ Several mouse models as well as clinical studies were subsequently conducted, emphasizing the implication of the proinflammatory cytokine IL‐17 in the pathogenesis of psoriasis and showing the advantageous usage of IL‐17 antagonist or of its receptor blockade on clinical symptoms of psoriasis.^66^ Furthermore*, Nfkbiz*‐encoded protein IκBζ contributes to the development of Th17 cells in mice.^67^ Previously, it was believed that IL‐6 and transforming growth factor‐β (TGF‐β) with the help of nuclear receptors RORγt and RORα were responsible for the development of Th17 cells.^68^ In 2010, Okamoto et al. demonstrated that the combined ectopic expression of IκBζ with RORγt or RORα in naive CD4^+^ T cells also led to remarkable induction of Th17 cells even in the absence of IL‐6 and TGF‐β.^67^ Moreover, *Nfkbiz* knockout mice were resistant to experimental autoimmune encephalomyelitis (a classical Th17‐dependent disorder).^69^ Overall, the data support that IκBζ is critically involved in IL‐17 mediated development of Th17 cells in multiple autoimmune disorders including psoriasis. Despite the existence of data obtained in synovial fibroblast during rheumatoid arthritis, it is noteworthy that Nfkbiz expression has not yet been studied in psoriatic arthritis, a frequent extra‐skin manifestation of psoriasis.

#### 1.7.1 Inducers of *NFKBIZ* in psoriasis

Numerous detailed studies were subsequently published, further pinning down the potential involvement of IκBζ in psoriasis and elaborating its mechanism of action. The cornerstone in this direction was the study by Johansen et al., in 2015, describing that IκBζ is a master regulator of psoriasis‐associated proteins such as CCL20, DEFB4, S100A7, IL‐8, IL‐19 and LCN2 in cultured human keratinocytes.^18^ The study provided evidence that IL‐17A is a strong inducer of *NFKBIZ* expression while TNFα has an insignificant impact on its expression. Lesioned psoriatic skin was found to express high level of IκBζ. Moreover, systemic and local deletion of IκBζ using siRNA results in either absence or reduction of psoriasis‐like skin lesions along with diminished expression of psoriasis‐related genes. This study delineated that IκBζ may be a better target than TNFα or IL‐17A to manage psoriasis, as psoriasis‐like skin inflammation was still occurring in the absence of TNFα and IL‐17A, whereas it was completely missing in the absence of IκBζ.^18^ Next, the impact of *NFKBIZ* knockdown was further demonstrated in human keratinocyte cell line, HaCaT. *NFKBIZ*‐deficient cells had a reduced expression of IL‐17A‐induced *DEFB4A, IL19* and *CSF3* genes even after co‐stimulation with TNFα. This finding emphasized that *NFKBIZ* is crucial for IL‐17A target genes.^19^ Reminiscent of IL‐17A, IL‐17F was also found to be associated with IκBζ‐mediated regulation of psoriasis‐associated genes in cultured human keratinocytes.^70^ Therefore, IκBζ has emerged as a key regulator of both IL‐17A and IL‐17F‐inducible psoriasis‐associated genes. Apart from the aforementioned inflammatory mediators, IL‐17A/F heterodimer which was recently identified to mimic both IL‐17A and IL‐17F was also found to regulate IκBζ‐mediated psoriasis associated genes.^71^ It is noteworthy that IL‐17A and TNFα were primarily considered as stimulants of IκBζ‐executed psoriasis‐like skin lesions; however, the search for additional IκBζ inducers was pursued. A possible rationale for this continuous search was the observation of elevated IκBζ mRNA levels in inflamed skin areas despite the global knockout of IL‐17A and TNFα in mice. These findings eventually underline the existence of IL‐17A/TNFα‐independent pathway accountable for IκBζ‐mediated downstream regulation of psoriasis‐associated genes.^18^

Taking forward this search, IL‐36α and IL‐36γ appeared to be also potent stimulators of IκBζ expression in both in vivo and in vitro studies. Thus, IL‐36‐induced IκBζ expression was demonstrated to be mainly supported by MyD88, NF‐κB and STAT3 activation. IκBζ‐deficient primary human keratinocytes and IκBζ knockout mice were also found to be prevented from IL‐36‐inducible psoriasis‐associated gene expression and psoriasis‐like dermatitis, respectively.^72^ These observations further validate the interest of modulating IκBζ for psoriasis therapy. Moreover, dsRNAs released from necrotic cells in skin and IL‐17A synergistically co‐stimulated IL‐36γ expression as well as other proinflammatory mediators. In this mechanism, IκBζ also accumulated and it was observed that IL‐17A and dsRNAs elevated IL‐36γ production through a p38 MAPK, NF‐κB and IκBζ‐dependent mechanism. Herein, a positive feedback loop is operated by NF‐κB‐induced IκBζ, which subsequently improves the NF‐κB binding to the IL‐36γ promoter.^56^ The studies by Müller et al. and Liu et al. highlighted the potential involvement of IL‐36γ in NF‐κB‐mediated IκBζ pathway in human keratinocytes.^56^, ^72^ Based on these studies, IL‐36γ might be positioned in a positive feedback loop with IκBζ, the latter being able to further up‐regulate IL‐36γ. In psoriasis patients receiving anti‐IL‐17A treatment, expression of *NFKBIZ* and *IL36G* are positively correlated. Furthermore, combined TNFα and IL‐17A stimulation led to an elevation in the expression of IL‐36γ which was found to be regulated by IκBζ.^73^ The downstream targets of IL‐17A‐ and IL‐36‐induced IκBζ, such as *Cxcl2*, *Cxcl5* and *Csf3*, were diminished after keratinocyte‐specific depletion of IκBζ. This abrogation did not alter T cell infiltration into the site of inflammation but was sufficient to suppress the recruitment of neutrophils and monocytes.^74^ Since IL36 has emerged as an important regulator of NF‐κB/IκBζ pathway in the pathogenesis of psoriasis but also in pustular psoriasis, a role for NF‐κB/IκBζ is highly probable in pustular psoriasis. These observations strongly suggest that IκBζ is abnormally expressed in psoriasis‐related cells and tissues. As its expression is regulated by several pro‐inflammatory mediators, IκBζ has emerged as a central modulator of NF‐κB‐mediated inflammatory response in psoriasis. Therefore, it is noteworthy that IκBζ inhibition has the potential therapeutic relevance to manage psoriasis symptoms, as illustrated by several in vivo and in vitro IκBζ knockout models. The graphical abstract provides an overview of the role of IκBζ in triggering inflammatory conditions under the influence of proinflammatory stimulus in psoriasis.

### 1.8 Inhibitors of IκBζ pathway

Considering that IκBζ plays an imperative role in the pathogenesis of psoriasis, the search for potent therapeutic agents inhibiting IκBζ is emerging. LPS and IL‐17 were considered as major inducers of IκBζ in psoriasis, and it was observed that dimethyl itaconate administration in psoriasis mice model suppressed LPS‐ and IL‐17‐induced IκBζ expression.^75^ Recently, tacrolimus, a T cell‐targeted immunosuppressant was examined on cultured human keratinocytes co‐stimulated with TNFα/ IL‐17A. This study reported that tacrolimus was successfully able to abrogate downstream targets of TNFα / IL‐17A‐induced IκBζ such as psoriasis‐associated genes *IL‐36γ, CCL‐20, IL‐1β* and *S100‐A9*.^76^ In addition to tacrolimus, dimethyl fumarate and secukinumab (an anti‐IL17A antibody) were also found to be protective in psoriasis by compromising IL‐17‐mediated induction of IκBζ.^46^, ^77^

### 1.9 IκBζ in cancer and other related‐pathologies

In addition to psoriasis, numerous studies have investigated the role of IκBζ in various types of cancers. For instance, IκBζ was found to be overexpressed in lymphoid cancers and activated B‐cell‐like subtype of diffuse large B‐cell lymphoma.^78^, ^79^ Increased transcription of *NFKBIZ* was also correlated with the occurrence of primary testicular and primary central nervous system lymphomas.^80^ Furthermore, the role of IκBζ in cancer was strongly validated when IκBζ was found to inhibit the transcriptional activity of Bcl3. In addition to Bcl3, IκBζ was also observed to inhibit the transcriptional activity of STAT3.^45^, ^81^ A study by Totzke et al. appraised the involvement of IκBζ in apoptosis and demonstrated that IκBζ repression induces resistance to apoptosis while its overexpression leads to cell death in human fibrosarcoma cells and breast carcinoma cells.^15^ The role of IκBζ was also shown in age‐related inflammatory disorders in both human and mouse.

The role of IκBζ in inflammatory disorders was further extended to osteoarthritis (OA); as in mice chondrocytes, IκBζ is overexpressed in response to IL‐1β. Furthermore, this overexpression caused upregulation in the levels of matrix‐degrading enzymes, thereby, associating IκBζ with cartilage destruction in OA. In addition to this, IκBζ was also found to be overexpressed in human OA cartilage as compared to undamaged cartilage. Similar observations were recorded in the experimental mice model for OA.^82^ Interestingly, IκBζ was also found to be involved in regulating IL17A and TNF‐induced transcription factor, ELF3 in synovial fibroblasts collected from rheumatoid arthritis (RA) and OA patients.^83^ In line with this, a recent study also supports that IκB‐ζ is involved in inflammation, senescence and oxidative stress in OA‐associated chondrocytes.^84^ The same group previously also described IκB‐ζ as a redox‐sensitive protein which partially contributes to the inflammation encouraged/favoured by LDHA‐induced ROS in OA‐associated chondrocytes.^85^ Additionally, the deletion of IκB‐ζ or STAT3 genes in murine epithelial cells was found to enhance apoptosis and early lymphocyte filtration which eventually leads to development of the Sjögren's syndrome‐like inflammation.^86^ In aged rat kidneys, up‐regulation of *Nfkbiz* was associated with increased macrophage infiltration. Furthermore, LPS‐treated aged rats were manifested with oxidative stress in the kidneys, whereas TGF‐β‐induced activation of kidney fibroblasts was found to be driven by *Nfkbiz*‐associated cytokines. These findings validate *Nfkbiz* involvement in age‐associated progressive renal fibrosis.^87^ Besides, IκBζ engages NF‐κB in fibroblasts and contributes to the production of chemoattractants in response to IL‐17, which are further responsible for the recruitment of neutrophils and monocytes to the site of inflammation.^88^ IκBζ was observed to play a crucial role in sepsis by elevating the expression of *Lcn2* which was also considered as predictor of mortality in septic patients.^89^ Moreover, NF‐κB‐mediated IκBζ signalling was also involved in initiating IL‐33‐dependent cytokine and chemokine production in bone marrow‐derived mast cells.^63^ The critical role of IκBζ in the pathogenesis of house dust mite–induced asthma was elucidated by Sundaram et al. This group described that IκBζ is involved in regulating inflammatory response in lung epithelium.^90^ Altogether, the abovementioned studies strongly suggest that IκBζ is critically involved in tumour expansion, apoptosis, recruitment of various inflammatory cells and secretion of both inflammatory cytokines and chemokines.

### 1.10 IκBζ in immune homeostasis and infections

Since IκBζ can positively alter the NF‐κB pathway and manipulate differentiation and recruitment of various immune cells, we speculate that IκBζ may be differentially expressed in bacterial infections and influences protective immune response. A recent study demonstrated that in the absence of IκBζ, the number of isolated lymphoid follicles and the absolute number of lymphocytes as well as their percentage fraction was significantly increased in the colonic *lamina propria* of mice. These observations hint toward the possible involvement of IκBζ in maintaining immune homeostasis in the gut.^91^ Interestingly, exposure of human blood monocytes to pneumococcal strain D39 successfully induces expression of IκBζ and downstream targets involved in host defense.^55^ Moreover, overexpression of IκBζ elevates the expression of IL‐6 and GMCSF in HEK293 cells while its knockdown diminishes mRNA expression of pneumococcus‐induced IL‐6 and GMCSF in monocytes. This study also demonstrated that expression of IκBζ is stimulated by TLR1/TLR2‐mediated p38 MAP kinase and NF‐κB.^55^ The immunoregulatory role of IκBζ was also exposed in T‐lymphocytes because IκBζ‐deficient T cells contribute to increment in peripheral effector/memory CD4+ cells and IFN‐γ‐producing CD4+ T cells. Furthermore, removal of IκBζ also revealed its significance in maintaining the plasticity and stability of Tregs.^92^ Furthermore, exposure of two gut commensals of low and high immunogenicity, *Bacteroides vulgates* and *Escherichia coli*, respectively, to bone marrow‐derived dendritic cells (BMDCs) leads to differential regulation of IκBζ expression. Of interest, secretion of IL‐6 and IL‐10 in response to infection was found to be dependent on IκBζ in BMDCs. This study also showed that the commensals stimulate TLR4 signalling which in response stimulates the secretion of Th‐17‐inducing cytokines in BMDCs.^93^ Moreover, IκBζ also upregulates Nlrp3 gene in bone marrow‐derived macrophages, thereby, playing crucial role in the activation of NLRP3 inflammasome.^94^ Interestingly, the potential regulatory role of *Nfkbiz* in the skin immunity was exposed when *Nfkbiz*‐deficient mice was found to spontaneously develop dermatitis along with expansion of *Staphylococcus xylosus* in the skin.^95^ Additionally, in Salmonella infection, IκBζ also emerged as an important preventive component by stimulating IgG secretion and Th1 differentiation.^96^ Ishiguro‐Oonuma et al. also observed that *Nfkbiz*‐deficient mice develop atopic dermatitis‐like lesions, and keratinocytes in these mice were less proliferative.^97^ Considering the significant contribution of IκBζ in the abovementioned inflammation‐mediated immune response, IκBζ seems to consequently be well suited to also fine tune the protective immune response under bacterial infections.

### 1.11 Future prospectives

Over the last decade, remarkable progress has been made for the management of psoriasis with neutralizing antibodies that target specific cytokines and immune cells. However, these therapies have difficult application routes as well as impose an economic burden for society considering their high cost. Also, the systemic utilization of neutralizing antibodies grounds for side effects such as upper respiratory tract infections, and can lead to progressive loss of efficacy owing to the development of anti‐drug antibodies. As a consequence, there is a need for the complementary development of a localized effective new therapy for psoriasis.^98^, ^99^ Moreover, Johansen et al. demonstrated that IκBζ inhibition is associated with reduced expression of psoriasis‐related genes and eventually provides protection against manifestations of psoriasis in both in vivo and in vitro models.^18^ Also, Mandal et al. successfully inhibited NFKBIZ by delivering small interfering RNA in skin with the help of ionic liquids and observed subsequent suppression of psoriasis‐related genes and signal.^100^ Hence, one can assume that IκBζ is a potential therapeutic target in IL‐17‐related inflammatory pathologies, including psoriasis. However, clinical inhibition of IκBζ is complicated as IκBζ lacks enzymatic activity making difficult the development of direct IκBζ inhibitors.^101^ Small molecule inhibitors or siRNA blocking the stimulation of IκBζ or hindering its downstream activity could be an alternative seducing pharmacological approach for psoriasis.

## 2 CONCLUDING REMARKS

The critical involvement of IκBζ pathway in inflammation and cell survival suggests its relevance to be used as a marker of psoriasis pathogenesis but also in other inflammatory disorders. Furthermore such observations indicate that therapeutically targeting IκBζ/NF‐κB pathway could be of interest in the management of these disorders.
